# Internal Impingement of the Shoulder: A Risk of False Positive Test Outcomes in External Impingement Tests?

**DOI:** 10.1155/2017/2941238

**Published:** 2017-08-20

**Authors:** Tim Leschinger, Christopher Wallraff, Dirk Müller, Matthias Hackenbroch, Henning Bovenschulte, Jan Siewe

**Affiliations:** ^1^Center for Orthopedic and Trauma Surgery, University Medical Center, Cologne, Germany; ^2^Cologne Center for Musculoskeletal Biomechanics, Medical Faculty, University of Cologne, Cologne, Germany; ^3^Department of Radiology, University Hospital of Cologne, Cologne, Germany; ^4^Center of Radiology Euskirchen, Euskirchen, Germany; ^5^PAN Clinic Cologne, Cologne, Germany

## Abstract

**Background:**

External impingement tests are considered as being particularly reliable for identifying subacromial and coracoid shoulder impingement mechanisms. The purpose of the present study was to evaluate if these tests are likely to provoke an internal shoulder impingement mechanism which, in cases of a pathologic condition, can lead to a positive test result.

**Method:**

In 37 subjects, the mechanical contact between the glenoid rim and the rotator cuff (RC) was measured quantitatively and qualitatively in external impingement test positions using an open MRI system.

**Results:**

Mechanical contact of the supraspinatus with the posterosuperior glenoid was present in 30 subjects in the Neer test. In the Hawkins test, the subscapularis was in contact with the anterosuperior glenoid in 33 subjects and the supraspinatus in 18. In the horizontal impingement test, anterosuperior contact of the supraspinatus with the glenoid was identified in 35 subjects.

**Conclusion:**

The Neer, Hawkins, and horizontal impingement tests are likely to provoke the mechanism of an internal shoulder impingement. A posterosuperior internal impingement mechanism is being provoked predominately in the Neer test. The Hawkins test narrows the distance between the insertions of the subscapularis and supraspinatus and the anterosuperior labrum, which leads to an anterosuperior impingement mechanism.

## 1. Introduction

The impingement tests of Hawkins and Neer as well as the horizontal impingement test are considered as particularly reliable for identifying subacromial and coracoid shoulder impingement syndromes and are routinely included in the clinical shoulder examination [1–6].

The tests were originally established to detect the pathology of a primary compressive shoulder impingement, as a direct result of compression of the rotator cuff tendons between the humeral head and the overlying anterior third of the acromion, the coracoacromial ligament, and the coracoid, with a positive test indicated by pain [3, 4]. Different studies proved the tests to be sensitive for subacromial pathologies but lacking specificity for specific injuries, since other conditions such as lesions of the glenoid labrum or arthritis can cause similar pain symptoms during testing [7, 8].

It has to be assumed that a pathological internal (undersurface) impingement, with fragmented tissue sheared and compressed between the humeral head and the glenoid, might also lead to a positive external impingement test and to misinterpretation of symptoms in the clinical shoulder examination. Whether these external impingement tests provoke the mechanisms of an internal shoulder impingement has not yet been conclusively elucidated in vivo. One study reported that subacromial, external impingement tests usually show negative results in patients with internal shoulder impingement; however, another report stated the opposite [9, 10]. For instance, Paley et al. reported that over 25% of throwing athletes show positive Neer and Hawkins tests on arthroscopy [10].

We hypothesized that external impingement tests provoke the mechanism of an internal shoulder impingement, which could result in positive test results in cases of pathology. Thus, the objective of this study was to qualitatively and quantitatively determine the mechanisms of an internal shoulder impingement that occur during Neer, Hawkins, and the horizontal impingement test, using an open MRI system.

## 2. Materials and Methods

**Figure 1:**
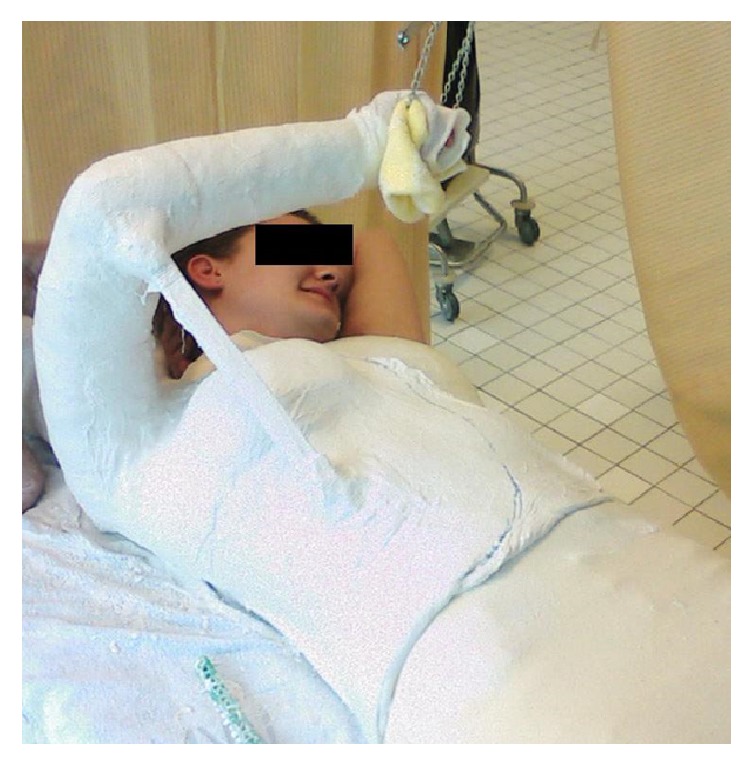
Application of the plaster splint.

The study initially included 38 healthy subjects. Magnetic Resonance Imaging (MRI) was carried out from a previous study [11]. One female subject was excluded because of inaccuracies in the available images; thus, the study was comprised of 19 males and 18 females. Average age of subjects was 24 years (range: 20 to 30 years), and all were right handed. Images were taken of the dominant shoulder. MRI was performed for all subjects using an open clinical 1.0T high-field MRI system (Panorama HFO, Philips Medical Systems, Best, NL). The open device enabled measurement of subjects with plaster splints (Figure 1) applied to the arm and thorax to maintain the following positions:

- Neutral position
- Hawkins test position (90° forward flexion of the shoulder with internal rotation of 15° and 90° flexion of the elbow)
- Neer test position (170° elevation of the shoulder with a stabilized patient's scapula)
- Horizontal impingement test position (90° abduction with 15° internal rotation of the shoulder)

All plaster splints were individually customized for each subject and positioned and applied with the help of a goniometer (Figure 1). The following imaging adjustments were made to perform the procedure: T1 weighted gradient echo imaging with parameters of TR = 38; TE = 6.9; FOV (field of view) = 170 × 170 mm; matrix = 320, with an approximate scan time of 5 minutes and 42 seconds. Each of the defined shoulder positions was captured in native T1 weighted 3D GRE sequence to ensure precise imaging data for the shoulder. The spatial resolution had a voxel size of 1 × 1.2 × 1.4 mm. The SAR (Specific Absorption Rate) was 4.1 Watt/kg.

Quantitative evaluation of all minimum distances and the closest proximity between the following pairs of structures were measured:

- The greater tuberosity and the glenoid
- The lesser tuberosity and the glenoid
- The bursal surface of the rotator cuff and the glenoid

**Figure 2:**
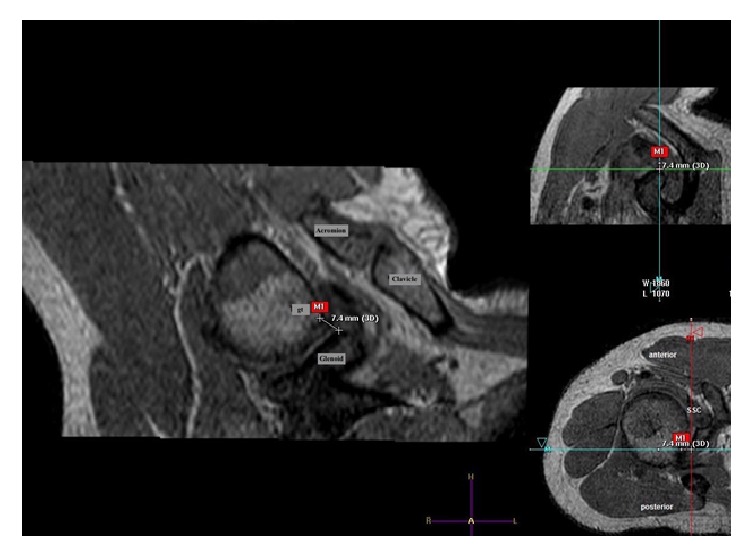
On the left: coronal plane MRI of subject's shoulder in Neer position with the smallest distance between the greater tuberosity and the glenoid rim (7.4 mm). Right above: sagittal, bottom: transverse sectional imaging.

Internal impingement was classified according to the extent of contact between the rotator cuff and glenoid. No contact was classified as grade 0. Grade 1 was assigned for contact without deformation of the rotator cuff and grade 2 when rotator cuff deformation was present. Using the acquired 3D image data records, distance measurements were performed by an experienced musculoskeletal radiologist on the workstation of the producer (Philips Extended Workspace, PMS, Best, NL), using implemented software for multiplanar reformatting (MPR) (Figure 2). The software enabled free reconstruction of the defined measuring points and thus helped create the required cutting planes. The absolute distances between the above-mentioned structure pairs were measured in the individual shoulder positions and compared with analysis of variance (ANOVA). Descriptive statistics such as percentages and averages were calculated. Subgroup analysis by gender was performed. The MCNemar test was used to compare the extent of rotator cuff contact with the acromion and coracoid. All analysis was performed using SPSS Statistics Version 22 (IBM Inc., Armonk, NY).

## 3. Results

**Figure 3:**
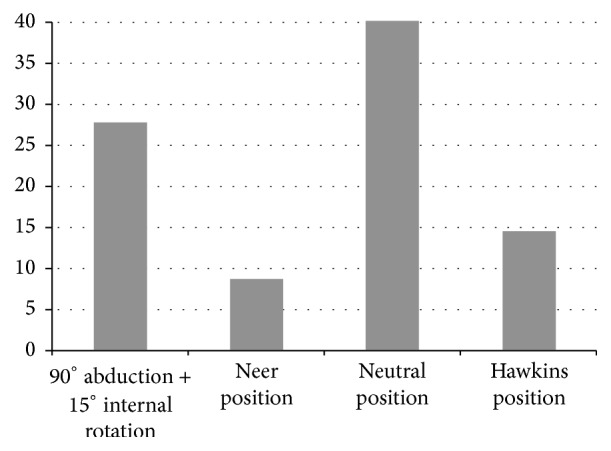
Minimum distance between the greater tuberosity and the glenoid in mm.

**Figure 4:**
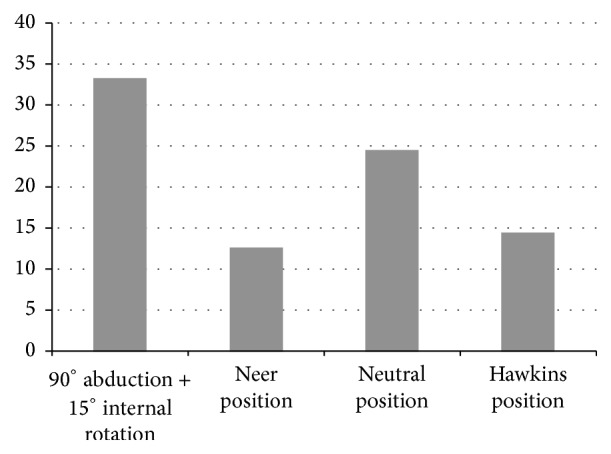
Minimum distance between the lesser tuberosity and the glenoid in mm.

Compared to the neutral position, the Neer and Hawkins tests resulted in significant narrowing of the minimum distance from the greater tuberosity to the glenoid (*p* < 0.001) (Figure 3). The minimum distance was 8.7. ± 1.7 mm in the Neer test, 25.5 ± 6.6 mm in the Hawkins test, and 27.8 ± 4.4 mm in the horizontal impingement test, significantly less than 40.2 ± 4.2 mm in the neutral position (*p* < 0.001). In addition, measurements in males were significantly greater in the Neer and Hawkins positions, with average mean distance 3.5 mm greater than in females (*p* < 0.001). The minimum distance between the glenoid rim and the lesser tuberosity was 12.7 ± 3.0 mm in the Neer test, and 14.6 ± 4.0 in the Hawkins test. Thus, the lesser tuberosity was significantly closer to the glenoid when performing the Neer and Hawkins tests than it is in neutral position (24.5 ± 5.2 mm). The distance increased to 33.3 ± 3.7 mm with the shoulder in the horizontal impingement test position (Figure 4).

After radiological grading of the image data by measuring the extent of contact between the rotator cuff and glenoid rim, mechanical contact of supraspinatus and the posterosuperior glenoid was evident in 30 subjects in the Neer test (25 without deformation, 5 with deformation) (*p* < 0.001). In the Hawkins test, superior and anterosuperior impingement with deformation (grade 2) of the supraspinatus was seen in four subjects; contact without deformation (grade 1) was observed in 14 (*p* < 0.001). In the horizontal impingement test, superior and anterosuperior contact was identified in 35 subjects (32 without and three with deformation) (*p* < 0.001) (Table 1). The surface of the infraspinatus touched the posterosuperior glenoid in 14 subjects in the Neer test, four in the Hawkins test, and two in the horizontal impingement test shoulders. None of these showed deformation of the tendon (Table 2).

Consistent with the proximity of the subscapularis insertion and the anterior glenoid, the subscapularis tendon and the glenoid showed mechanical contact in the Neer and Hawkins tests (*p* < 0.001). In the Neer test, deformation of the tendon at the anterosuperior labrum occurred in one subject and mechanical contact in 24 subjects. In the Hawkins test, the subscapularis was in contact with the anterosuperior glenoid in 33 subjects (four with deformation, 29 without) (Table 3).

## 4. Discussion

The present study examined the in vivo mechanism of internal shoulder impingement during the Neer, Hawkins, and horizontal impingement test. In the study population, we found an increased risk of a posterosuperior impingement (PSI) mechanism being provoked in the Neer test, when the supra- and infraspinatus press against the posterosuperior glenoid and labrum. The Hawkins test narrowed the distance between the insertions of the subscapularis and supraspinatus and the anterosuperior labrum, leading to mechanical contact of the tendons and a potential provocation of an anterior internal impingement (ASI). In the horizontal impingement test, anterosuperior contact of the supraspinatus with the glenoid was identified in 35 subjects (32 without and three with deformation). As a consequence, it has to be assumed that a pathologic internal impingement can lead to positive Neer, Hawkins, and horizontal impingement tests.

As yet, there was no agreement whether the external impingement tests of Neer and Hawkins are usually negative in patients with internal impingement [9, 10]. A study of 41 professional throwing athletes reported that over 25% exhibited positive Neer and Hawkins tests [10]. In the present study, the distance from the glenoid rim to the greater tuberosity narrowed significantly during the classic external impingement tests. This was especially evident in the Neer test, where the minimum distance measured 8.7. ± 1.7 mm versus 40.2 ± 4.2 mm measured in the neutral position (*p* < 0.001). As a consequence, mechanical contact of supraspinatus with the posterosuperior glenoid was observed in 30 shoulders in the Neer test position (25 without and five with deformation). This type of internal impingement (PSI), as described by Jobe [12], can usually be provoked when the supraspinatus contacts the glenoid and labrum in the mid-acceleration phase of the throwing movement [9, 13–16]. Typical findings for PSI include partial thickness rotator cuff tears on the articular side and concomitant posterosuperior or posterior labral injuries [9, 14]. An ASI mechanism of the supraspinatus was identified in 18 shoulders (four with deformation and 14 without) in the Hawkins position and in 35 shoulders (32 without deformation and three with) in the horizontal impingement test position. In addition, the Hawkins test narrowed the distance between the glenoid and the lesser tuberosity. This is consistent with the localisation of the insertion of subscapularis and the observed contact of the subscapularis and the anterosuperior labrum in 24 shoulders found in these test positions. In these cases, it is important to note the location of the impingement mechanism, that is, between the anterior humeral head and the anterosuperior glenoid and labrum, which can lead to an ASI [15]. Lesions of the long head of the biceps (LHB), the biceps pulley, and the rotator cuff have been associated with ASI. Tears of the undersurface of the anterior supraspinatus tendon have also been identified [17, 18].

The presented findings help to explain why positive test outcomes in external impingement tests seem to allow for a variety of possible pain mechanisms, which in turn makes it hard to provide pathoanatomic diagnosis on the basis of positive testing. The results of this study indicate why diagnostic labels based on clinical examinations are prone to failure and why it is difficult to accurately classify patients into subgroups for clinical decision-making processes [19]. Although the investigated tests are frequently used in clinical examination to detect and provoke a primary, compressive subacromial and coracoid shoulder impingement [3, 4], physicians should also consider internally located pathologic mechanisms in the joint with related concomitant shoulder injuries as cause for positive external impingement test results. Therefore, the presented findings enable a thorough understanding of the underlying pathophysiological and varying biomechanical mechanisms, which will help physicians to more accurately interpret findings of the relevant clinical shoulder examination.

However, the present study has some limitations. One is that only healthy volunteers were enrolled. More research in symptomatic shoulders (e.g., cases of rotator cuff tear) is necessary to enable intersubject comparison of rotator cuff contact and bony distances. It is also important to note that our study subjects were recumbent. It is possible that gravity could have pulled the humeral head slightly posterior compared to a standing position.

This in turn could have led to changes in the three-dimensional location of the shoulder joint, which might have impacted our measurements. In addition, we were unable to capture dynamic imaging. Further investigations and comparisons using kinematic MRI to identify internal impingement pathology would be useful.

## 5. Conclusion

The external impingement tests of Neer and Hawkins and the horizontal impingement test are likely to provoke internal impingement mechanisms of the shoulder. A PSI mechanism is being provoked predominately in the Neer test, when the supra- and infraspinatus push against the posterosuperior glenoid and labrum. The Hawkins position narrows the distance between the insertions of the subscapularis and supraspinatus and the anterosuperior and superior labrum, which leads to mechanical contact of the tendons and a potential provocation of an ASI mechanism.

## Figures and Tables

**Table 1:** Mechanical contact between the supraspinatus and the glenoid labrum (in %).

Mechanical contact	Shoulder position			
	90° abduction + 15° internal rotation “horizontal impingement test”	170° abduction “Neer test”	Neutral position	90° flexion, + 15° internal rotation “Hawkins test”
No impingement	5.4	18.9	100.0	51.4
Impingement without deformation	86.5	67.6	0	37.8
Impingement with deformation	8.1	13.5	0	10.8

Overall	100.0	100.0	100.0	100.0

**Table 2:** Mechanical contact between the infraspinatus and the glenoid labrum (in %).

Mechanical contact	Shoulder position			
	90° abduction + 15° internal rotation “horizontal impingement test”	170° abduction “Neer test”	Neutral position	90° flexion + 15° internal rotation “Hawkins test”
No impingement	94.6	62.2	100.0	89.2
Impingement without deformation	5.4	37.8	0	10.8
Impingement with deformation	0	0	0	0

Overall	100.0	100.0	100.0	100.0

**Table 3:** Mechanical contact between the subscapularis and the glenoid labrum (in %).

Mechanical contact	Shoulder position			
	90° abduction + 15° internal rotation “horizontal impingement test”	170° abduction “Neer position”	Neutral position	90° flexion, + 15° internal rotation “Hawkins position”
No impingement	86.5	10.8	100.0	32.4
Impingement without deformation	13.5	78.4	0	64.9
Impingement with deformation	0	10.8	0	2.7

Overall	100.0	100.0	100.0	100.0
